# Governance of Eswatini Apparel Regional Value Chains and the Implications of Covid-19

**DOI:** 10.1057/s41287-021-00383-3

**Published:** 2021-03-10

**Authors:** Giovanni Pasquali, Shane Godfrey

**Affiliations:** 1 The Global Development Institute, University of Manchester, Arthur Lewis Building, Room 2.037, Oxford Road, Manchester, M13 9PL UK; 2 Institute of Development and Labour Law, University of Cape Town, Cape Town, South Africa

**Keywords:** Regional value chains, Apparel, Governance, Upgrading, Covid-19, Customs data, Eswatini, South Africa

## Abstract

There is a growing literature on the impact of Covid-19 on commercial and labour conditions at suppliers in apparel global value chains (GVCs). Yet much less is known about the implications for suppliers operating in regional value chains (RVCs) in the global South. In this article, we focus on Eswatini, which has grown to become the largest African manufacturer and exporter of apparel to the region. We draw on a combination of firm-level export data and interviews with stakeholders before and after the Covid-19 lockdown to shed light on the influence of private and public governance on suppliers’ economic and social upgrading and downgrading. We point to the coexistence of two separate private governance structures: the first characterised by direct contracts between South African retailers and large manufacturers (*direct suppliers*); the second operating through indirect purchasing via intermediaries from relatively smaller producers (*indirect suppliers*). While direct suppliers enjoyed higher levels of economic and social upgrading than indirect suppliers before Covid-19, the pandemic reinforced this division, with severe price cuts for indirect suppliers. Furthermore, while retailers provided some direct suppliers with support throughout the crisis, this was not the case for indirect suppliers, who remain comparatively more vulnerable. In terms of public governance, the negative consequences of the lockdown on firms’ income and workers’ livelihoods have been compounded by the state’s ineffective response. Our paper contributes to the research on RVCs in the global South, enhancing our understanding of how different governance structures and external shocks affect firms’ and workers’ upgrading and downgrading prospects.

## Résumé

La publications à propos l’impact de la COVID-19 sur les conditions commerciales et du travail chez les fournisseurs des chaines de valeur globales (en anglais : Global Value Chains, GVCs), dans le secteur de l’habillement, est en croissance. Cependant, on connait beaucoup moins à propos les implications de la COVID-19 sur les fournisseurs qui opèrent au niveau des chaines de valeur régionales (en anglais : Regional Value Chains, RVCs) dans les pays du Sud. Dans cet article, nous nous concentrons sur Eswatini, qui est devenu le plus grand producteur Africain et exportateur d’habillement dans la région. Combinant des données collectés au niveau de l’entreprise sur ses exportations, et des entretiens avec plusieurs partis prenants avant et après le confinement COVID-19, nous illustrons l’influence de la gouvernance publique et privée sur la revalorisation et dévalorisation sociale et économique des fournisseurs. Nous attirons l’attention à la coexistence de deux structures séparés de gouvernance privée : la première structure est caractérisée par des contrats directs entre les détaillants de l’Afrique du Sud, et les gros producteurs (fournisseurs directs) ; la deuxième structure est assurée par les achats indirects chez les producteurs relativement plus petits (fournisseurs indirects) par des intermédiaires. Alors que les fournisseurs directs bénéficiaient de des niveaux de revalorisation économique et sociale plus élevés même avant la COVID-19, la pandémie a renforcé leur séparation par rapport aux fournisseurs indirects ; ces-ci ont plutôt subi une forte réduction des prix payés pour leur production. D’ailleurs, les détaillants ont soutenu les fournisseurs directs pendant la crise, mais ceci n’a pas été le cas pour les fournisseurs indirects, qui demeurent plus vulnérables. En ce qui concerne la gouvernance publique, les conséquences négatives du confinement sur le revenu des entreprises et sur la subsistance des travailleurs ont été aggravés par les interventions inefficaces de l’état. Cet étude contribue à la recherche sur les RVCs dans les pays du Sud, améliorant notre compréhension de comment les différentes structures de gouvernance et les chocs externes touchent les perspectives de valorisation et dévalorisation des entreprises et des travailleurs.

## Introduction

The outbreak of the Covid-19 pandemic is having major implications for the governance of apparel global value chains (GVCs). The actions of international retailers who, in a number of cases, have delayed or cancelled orders, and refused to pay for ongoing production and shipments, has placed immense pressure on suppliers (Anner 2020a). This situation has been compounded by temporary lockdowns by governments to constrain the spread of the virus. The combination of retailers’ and governments’ actions has led to a number of negative consequences for suppliers’ income and the welfare of their labour force, including widespread closures, downsizing and unemployment. Limited state support has meant that these negative consequences have been especially severe in developing countries, where state support for manufacturers is limited (Teodoro and Rodriguez 2020). In a concept central to GVC literature (Barrientos et al. 2011), the Covid-19 pandemic has therefore translated into economic and social downgrading for suppliers and workers in the global South.

While increasing attention is being paid to the implications of the pandemic for firms in the global South supplying retailers in Europe and the USA (Devnath 2020; Kelly 2020; Mirdha 2020), we know less about its impact on regional production networks connecting buyers and suppliers in developing countries. In this context, scholars have used the concept of regional value chains (RVCs) to indicate inter-firm networks coordinated and governed by lead firms ‘primarily operating within one world region’ (Barrientos et al. 2016b, p. 1280; Krishnan 2018; Pasquali 2021a). This research has emerged against a backdrop where the value of South–South trade overtook North–South trade in the 2010s, with intraregional commerce accounting for a large share of the global South’s improved trade performance (Horner and Nadvi 2018; McKinsey and Co 2019; Pasquali 2021b).

Given the growing importance of regional trade (EIU 2020; Pasquali et al. 2020), this article aims to examine the interaction of both private and public governance in shaping suppliers’ economic and social up- and downgrading in RVCs (Alford and Phillips 2018). Moreover, by focussing on the periods both before and during a large exogenous shock—i.e. the Covid-19 pandemic—our paper provides a unique comparative account of RVCs’ adaptive responses to major shocks.

We focus on the Kingdom of Eswatini (henceforth Eswatini), a landlocked country in Southern Africa with a population of 1.1 million people and a member of the Southern African Customs Union (SACU) with South Africa, Botswana, Namibia, and Lesotho. Since 2014, Eswatini has grown to become the largest regional exporter of apparel to Sub-Saharan Africa, with over 95% of its export share going to South Africa. With a focus on Eswatini’s apparel sector, we address three main questions: (1) How do South African lead firms govern their interaction with Eswatini’s apparel suppliers? (2) What have been the implications of such governance for suppliers’ economic and social upgrading before and during the Covid-19 pandemic? And (3) what has been the role of the state’s public governance in shaping the latter? To answer these questions, we draw on interviews conducted with suppliers and key stakeholders before and during the Covid-19 pandemic. Importantly, we complement this evidence with information from a unique dataset of transaction-level export data covering the 2017–20 period.

In the first part of the paper, we focus on question (1) and provide an overview of the Eswatini apparel industry in relation to its participation in RVCs connected to South African retailers. Notably, we point to the coexistence of two separate governance structures: the first characterised by direct contracting between lead firms and comparatively large suppliers (i.e. direct suppliers); the second operating through indirect purchasing via intermediaries known as design houses, which manage the interaction between retailers and a set of relatively small suppliers (i.e. indirect suppliers). This division, we argue, has important implications for the economic and social upgrading of local producers, with direct suppliers displaying significantly higher scores on both measures.

In the second part of the paper, we answer questions (2) and (3) by focussing on the effect of the Covid-19 lockdown on suppliers’ up- and downgrading, and on how public governance interventions have shaped such impact. Our conclusions suggest that the impact of the lockdown on the Eswatini apparel industry has been severe in terms of firms’ income and workers’ employment and earnings, therefore resulting in economic and social downgrading. The consequences have been relatively worse for indirect suppliers, which experienced significant price cuts of about 14%. Conversely, all (but one) South African buyers honoured their contractual agreements with Eswatini direct suppliers and, in a few cases, provided them with financial support. Direct suppliers were also more successful in differentiating into the production of PPE clothing, further cushioning the impact of the lockdown.

This evidence differs in degree from the situation in Bangladesh’s apparel GVCs, where many large European and American buyers refused to honour their contractual obligations, with catastrophic consequences for the local industry (Anner 2020a). Nevertheless, as we argue, the situation in the Eswatini apparel sector remains critical and demands a concerted effort from firms (buyers and suppliers), workers’ representatives and the government. Despite ongoing negotiations between the government, the main union in the sector and employers, as of July 2020, Eswatini had yet to implement any Covid-related support measures.

The remainder of the paper is structured as follows. “Governance and Upgrading in Apparel GVCs and RVCs” discusses the literature and main concepts. “Data and Methodology” describes the data and methodology. “Eswatini Apparel RVCs Before Covid-19” presents an analysis of the value chain governance and firms’ upgrading in the period before Covid-19. “Eswatini Apparel Value Chain During Covid-19” illustrates the impact of the pandemic on firms’ economic and social up- and downgrading, and further describes the role of public governance in shaping it. Finally, “Conclusion” discusses the results and concludes.

## Governance and Upgrading in Apparel GVCs and RVCs

Arguably, more has been written on apparel than on any other value chain. As a labour-intensive sector with relatively inexpensive technological requirements, apparel has been identified as a key industry for kick-starting industrialisation in the global South, through participation in GVCs serving retailers in developed countries (Bair and Gereffi 2003; Gereffi and Frederick 2011). This literature has focussed on how large retailers in the USA and Europe—i.e. ‘lead firms’—manage their interactions with suppliers in developing countries, a process referred to as ‘private governance’ (Gereffi et al. 2005). Gereffi’s (1999, p. 40) and Staritz’s (2011) seminal works on East Asia’s and Southern Africa’s apparel sectors point to contrasting ways of governing apparel value chains. In the first, lead firms own and provide the inputs, primarily designs and fabric, for assembly by garment sewing plants that simply cut the fabric and assemble the garments (termed cut, make and trim (CMT) suppliers). In the second, suppliers source the inputs themselves according to buyers’ specifications, effectively owning the garment that they package and sell to retailers (known as free-on-board (FOB) manufacturing). It has been argued that the movement from CMT to FOB manufacturing is critical for firms and countries to reap the economic benefits of participation in GVCs (Gereffi and Frederick 2011). The process of improving products and processes, and taking on new production functions ‘to increase the benefits or profits deriving from participation in GVCs’ (Barrientos et al. 2011, p. 323) has been termed ‘economic upgrading’. Conversely, ‘economic downgrading’ relates to participation in GVCs that leads to a drop in value-adding gains.

Another strand of GVCs literature has been concerned with labour conditions at suppliers in apparel GVCs (Anner 2015; Godfrey 2015; Bair 2017). One focus of this research has been whether and how economic benefits from participation in GVCs translate into welfare gains or losses for the labour force—respectively, referred to as ‘social up- or downgrading’ (Barrientos et al. 2011). On the one hand, scholars have paid increasing attention to the impact of lead firms’ codes of conduct and audits on workers in manufacturing plants, which falls under the rubric of private governance. On the other hand, investigation of labour conditions has also seen greater consideration for actors ‘outside’ the value chain—in particular the state and civil society organisations (Mayer and Phillips 2017). The concepts of public and social governance have been used to analyse how, respectively, the state and civil society influence the formation and organisation of value chains (Gereffi and Lee 2016; Alford and Phillips 2018).

Having recognised that civil society campaigns in developed countries played a critical role in pushing lead retailers to impose corporate codes of conduct on their suppliers (Barrientos and Smith 2007; Lund-Thomsen and Lindgreen 2014), research on social upgrading sought to understand how public and social governance in the localities where suppliers are based contributed to improved wages and working conditions. Generally, the results have indicated only a limited impact on labour conditions by private governance mechanisms combined with public and social governance: weak labour market institutions in developing countries and the reliance on foreign investments have forced governments to seek compromises, with private governance not able on its own to make a significant impact (Amengual 2010; Locke 2013; Phillips 2013; Mayer and Phillips 2017).

Cutting across aspects of economic and social upgrading, much of the abovementioned literature has examined value chains linking retailers in the global North to suppliers in the global South (Horner and Nadvi 2018; Pasquali 2021b). Value chains oriented towards Southern end-markets have been overlooked until relatively recently (Barrientos et al. 2016a; Horner 2016). Filling this gap is critical considering the unprecedented growth of South–South trade, together with the rise of RVCs (UNCTAD 2015; Mohanty et al. 2019).

While a large proportion of international apparel trade remains North–South, RVCs in the South have been growing (Pickles et al. 2015). This is particularly the case in Southern Africa, where clothing manufactured in Eswatini, Lesotho, Mauritius, and Madagascar have increasingly flowed into South Africa (Morris et al. 2016; Godfrey 2015; Pasquali et al. 2020). Benefitting from the Multi-Fibre Arrangement (MFA) up to 2004 and the Africa Growth and Opportunity Act (AGOA) from 2001, the industry first developed through ‘triangular sourcing’ in GVCs involving manufacturers headquartered in East Asia serving lead buyers in Europe and the USA via CMT facilities located in the region (Gibbon 2008). Between 2005 and 2018, however, intra-Africa trade in apparel grew from a share of 8% to about 35% of the region’s total output (Pasquali et al. 2020). This trend has been underscored by the lead role of South African brands largely catering to the South African market. Three main factors have been critical to the development of apparel RVCs in the region: (i) duty and quota free trade under the Southern African Customs Union (SACU) and Southern African Development Community (SADC) rules of origin; (ii) favourable investment polices attracting South African investors to Lesotho and Eswatini, and Mauritian ones to Madagascar; and (iii) more comprehensive labour legislation in South Africa, resulting in significantly higher minimum wages compared to other countries in the SACU and SADC areas (Pasquali et al. 2020; Morris and Staritz 2014).

In the context of apparel RVCs in Southern Africa, existing studies have focussed on different governance and upgrading outcomes across firms participating in GVCs versus RVCs. This literature categorises regional suppliers depending on their origin and end-markets as follows: (1) transnational investors, characterised mostly by Asian entrepreneurs interested in leveraging the country’s duty-free access to the US market under AGOA; (2) regional investors targeting the South African market; and (3) the so-called ‘diaspora investors’, who developed strong domestic linkages while maintaining global linkages favoured by their foreign descent (Staritz 2011; Morris et al. 2016).^1^ According to this research, these three categories have led to different upgrading outcomes, with regional investors operating smaller plants, and focussing on smaller orders of more complex products, while also facilitating more knowledge transfer and training of workers (Morris et al. 2016).

While this literature provides an important comparative analysis across suppliers serving GVCs and RVCs, it does not tell us much about potential differences within RVCs. Are there different governance structures within RVCs? Do regional suppliers vary in terms of economic and social upgrading prospects, and if so, why? The Covid-19 pandemic has sharply foregrounded these questions. Anner (2020a), for example, has shown how European and American retailers in GVCs failed to honour their contractual obligations with suppliers in Bangladesh, leading to widespread closures and labour retrenchments. Similar dynamics have also been reported in Sri Lanka, Cambodia and Pakistan (Narim 2020; Toppa 2020). No similar research has, however, been conducted on how the pandemic has impacted RVCs in the global South. Furthermore, few studies have explored how private and public governance interact to shape suppliers’ economic and social upgrading (or downgrading) in response to exogenous shocks (Baldwin and Evenett 2020). In the next sections, we therefore provide an important and unique contribution to the literature by highlighting how different governance structures shape the way shocks affect firms’ and workers’ up- and downgrading prospects in RVCs.

## Data and Methodology

In this study we focus on Eswatini’s apparel sector, drawing on three data sources. First, semi-structured interviews conducted in July 2019 with all 20 apparel manufacturers in the country. These include 15 small- to medium-sized plants (with less than 1000 employees) operating mostly via *design houses*, and five large suppliers (with over 1000 employees) operating through direct contracts with South African retailers. Interviews conducted before the Covid-19 crisis are used to illustrate the structure and governance of Eswatini’s apparel value chain, paying particular attention to the commercial and social implications for suppliers operating through direct contracts with regional lead firms (direct suppliers), and suppliers selling into RVCs through intermediaries (indirect suppliers). For this purpose, we consider a set of economic structural indicators, such as firm production capacity and number of employees; basic firm-level indicators of value addition, including functions performed and average mark-ups; and private governance indicators, including the internal structure of the firm and the adoption of private standards. Furthermore, drawing on previous studies on social upgrading, we assess differences in terms of labour conditions and entitlements (Barrientos and Smith 2007), including wages, share of workforce that is permanent, union organisation in the firm, access to healthcare facilities, and the presence of regular social audits (Barrientos et al. 2011; Pasquali 2021a). To the extent that women make up over 90% of the sectoral workforce, we also looked at whether there are gender discrimination policies and committees in the factories (Barrientos 2019). Data on labour were collected from firms’ management and further triangulated with information from interviews with a limited number of workers (i.e. one to two workers per firm in 15 of the 20 firms), who were approached through the Amalgamated Trade Union of Swaziland (ATUSWA) in July 2019.^2^

Second, to evaluate the impact of Covid-19, we examine transaction-level customs data for apparel exports collected by the Eswatini Revenue Authority over the 2017–20 period (up to April, 30th 2020). Every observation in the dataset corresponds to an export transaction with information on quantity (kg), real value in ZAR, date of transaction, unique identifiers for exporting and importing firms, country of destination, and the World Customs Organization’s Harmonized System (HS) 8-digit code identifying the traded product. For the purposes of this paper, we consider only transactions that took place in the months of March and April 2017, 2018, 2019 and 2020. This is because apparel is a very seasonal industry, and it would make little sense to compare different months across the year. To capture the impact of the first lockdown (from March 2020), we therefore calculate moving averages for the period 2017–19 and compare them to their respective indicators for 2020. We consider four main indicators at the aggregate and the firm level, which are used as indicators of economic up- and downgrading:

### Total exported value

We compute the total exported value at the sector level for apparel (including HS-61, -62 and -63) and compare it before and during the Covid-19 lockdown. Furthermore, we calculate average exported values at the firm level. Changes in a country’s and a firm’s total and average exported value in a given sector are indicators of economic up- and downgrading (Bernhardt and Milberg 2012).

### Unit Values

We use the common practice of measuring product quality using unit values (Schott 2004; Hallak 2006). An increase or decrease in unit values has been used as an indicator of economic upgrading and downgrading, respectively (Bernhardt and Milberg 2012; Bernhardt and Pollak 2016; Van Assche and Van Biesebroeck 2018). This is calculated as the natural log of the transaction’s real value divided by the quantity exported (Pasquali 2021b).

### Product Diversification

We calculate a firm’s average number of exported products at the 6-digit HS level and within the broader apparel HS levels (61–63).^3^ An increase in ‘related product diversification’ across products within the same sector has been associated with increased business performance, and is therefore also an indicator of economic upgrading (Hitt et al. 1997; Chang and Wang 2007).

Our third data source takes the form of a second round of questionnaires distributed to firms online in June 2020 to triangulate the outcome of the data analysis at the firm level. We sent a short questionnaire to all 20 suppliers interviewed over the previous year. We received eight responses. Because of the relatively small number of responses, these data are not fully representative. We therefore refrain from drawing final conclusions from the online questionnaires. Instead, responses are considered complementary to the evidence emerging from customs data and are used to shed further light on aspects underpinning the impact of the Covid-19 crisis on firms relative to their economic performance and labour situation. A copy of the questionnaire is available in the Appendix.^4^

## Eswatini Apparel RVCs Before Covid-19

**Fig. 1:**
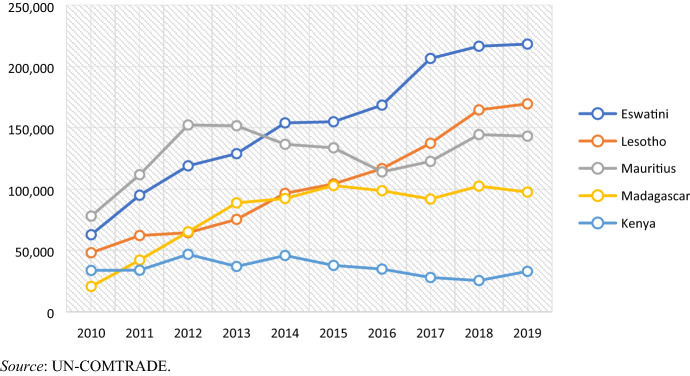
Intra-Africa apparel exports by main apparel exporting countries: 2010–18 (US$000)

Apparel represents a crucial source of income for Eswatini’s economy, employing over 22,000 people and accounting for 10.5% of the country’s 2019 exports. Despite its small size relative to global apparel production, the country is a crucial player in RVCs feeding into the South African market (Staritz et al. 2016). As shown in Fig. 1 (data from Table 1, Appendix), Eswatini is the largest apparel exporter to the region and the one that experienced the steepest overall growth in terms of regional exports. In 2019, it accounted for 33% of all intraregional apparel exports in Sub-Saharan Africa, followed by Lesotho (26%), Mauritius (21%) and Madagascar (14%).^5^

**Fig. 2:**
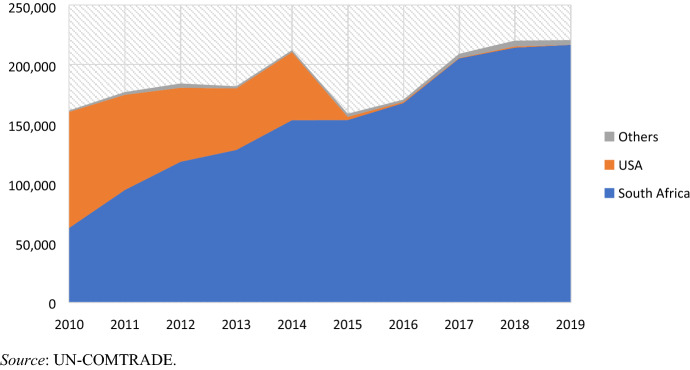
Eswatini’s apparel export share by destination (US$000). (Color figure online)

An extensive literature has discussed the emergence of the apparel sector in Eswatini, driven by Taiwanese FDIs in the early 1990s taking advantage of unused MFA quotas to access the US market (Staritz and Morris 2012; Pickles et al. 2015). Despite the phasing out of the MFA, the African Growth and Opportunity Act (AGOA) effectively extended the country’s preferential access to the US market starting from 2001 (Morris et al. 2011; Staritz 2011). However, in the past 15 years, Eswatini’s apparel industry has increasingly strengthened its linkages to RVCs via South African investments attracted by lower labour costs and Southern African Customs Union (SACU) preferential trade regulations (Morris et al. 2011,2016). The country’s exclusion from the AGOA in 2014–15 further accelerated its shift towards the South African market (Pasquali et al. 2020). By 2015, all overseas exporters had successfully shifted to supply large South African retailers, including major brands such as Mr Price, the Foschini Group (TFG), Pepkor, Edcon, Woolworths and Truworths.^6^ In addition, following the renewed interest of South African retailers, a number of new suppliers (including a large investment moving from Lesotho) increased the country’s total output (Pasquali et al. 2020). Despite Eswatini regaining its AGOA status in 2018, as of today, exports to the US have not resumed and South Africa remains the main market for roughly 95% of the country’s clothing output, with most of the remaining 5% destined to other countries in the SADC region (Fig. 2, data in Table 2 in the Appendix).

### Private Governance: Direct vs Indirect Suppliers

The vast majority of Eswatini’s apparel exports goes to South Africa. However, the governance of the value chain varies. On the one hand, six to eight firms have established direct interactions with South African retailers (we refer to these as *direct suppliers*),^7^ which implies the presence of a contract linking the manufacturer to the final retailer, as well as ongoing personal engagement. On the other hand, 12–14 firms operate indirectly through design houses (we refer to these as *indirect suppliers*), which act as intermediaries for South African retailers. In this case, the suppliers never interact with the final retailers and their awareness of the order’s final destination is apparent only from the tag applied on the finished clothing.^8^ Instead, their relationship operates through third-party agencies that provide the suppliers with the designs and fabric, and deliver the final products to the retailers. The design houses operating n Eswatini are all based in South Africa. Our data indicate that at least six design houses were active in Eswatini in 2019, with linkages to three large South African retailers—Pepkor, Mr Price and Edcon, i.e. the major discount retailers. Conversely, brands such as TFG, Woolworths and Truworths appeared to operate largely via direct contracts.^9^

**Fig. 3:**
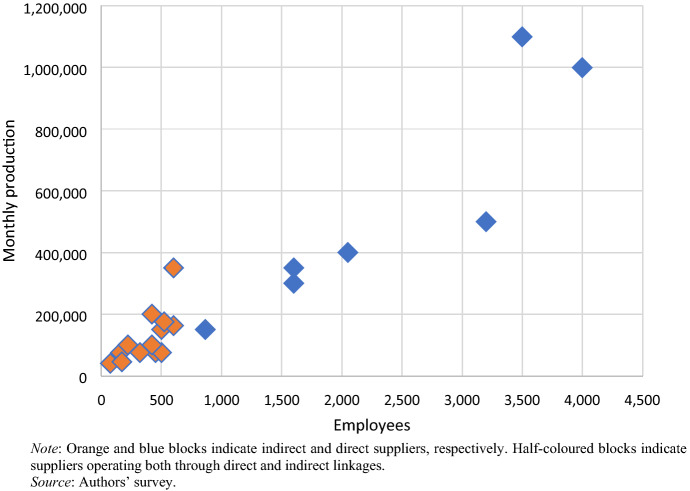
Direct and indirect apparel suppliers by number of employees and monthly capacity (2019). (Color figure online)

An important difference between direct and indirect suppliers is the significantly larger size of the former. Direct suppliers employ an average of 2176 workers and produce an average of 490,000 pieces per month, whereas indirect suppliers employ only 363 workers on average and produce 120,000 pieces per month (Fig. 3). In addition, our pre-Covid-19 survey points to significantly different implications for the economic and social upgrading of direct and indirect suppliers. We examine these below.

### Differences in Economic Upgrading Before Covid-19

**Fig. 4:**
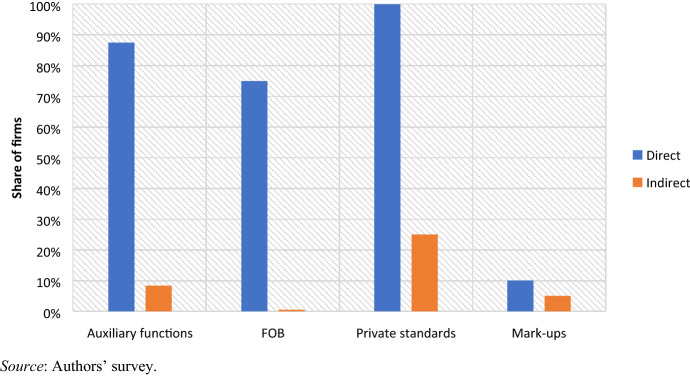
Economic upgrading among direct and indirect suppliers (2019)

In terms of economic upgrading, we identify four major implications for suppliers which differ according to whether they contract directly with retailers or indirectly via design houses (Fig. 4).

First, 75% of direct suppliers operate as FOB manufacturers, while all indirect suppliers in Eswatini perform exclusively CMT operations. To the extent that FOB firms directly source and own production inputs (including fabrics), their yearly turnover is considerably greater than CMT firms. This has important repercussions in terms of public revenues, in that FOB suppliers contribute significantly more to the public purse through direct and indirect taxation. The manager for investment promotion at the Eswatini Investment Promotion Authority (EIPA) argued:

There isn't much direct benefit from CMT factories, beyond employment. FOBs pay taxes because their profits can be monitored, unlike the CMTs because they tell you that, “We don’t own these products”, to the extent that you don’t even know how their accounts are structured. If you do not own the product, it does not appear in your balance sheet and inventory. (Mbabane, 8 July 2019)

Second, 87% of direct suppliers are found to perform at least one auxiliary function (beyond cutting and sewing), including laundering, printing and embroidering, whereas this is the case for fewer than 10% of indirect suppliers. This suggests that direct suppliers are likely to capture a greater share of value added in the production process.

Third, all direct suppliers reported being subject to a number of private standards and certifications, including the SEDEX audits (four firms, two of which reported using the ETI base code), WRAP (one firm), ISO-9001 (one firm), and a number of buyers’ internal audits, such as the Woolworth’s gold-silver-bronze-red ranking system (WHL 2019). These audits benchmark firms based on a wide range of measures beyond production, including health and safety, and labour and environmental standards. Conversely, only 25% of indirect suppliers reported formal auditing, emphasising instead how design houses regularly send their staff to monitor product quality only.

Fourth, direct suppliers tend to specialise in more complex clothing with a higher fashion content (including women’s wear). This translates into comparatively higher unit values and mark-ups. Data on mark-ups have been disclosed only by a handful of firms, with considerable variations. Nevertheless, while direct suppliers reported mark-ups in the range of 6% to 14%, indirect suppliers reported working on margins as low as 2% and up to 8%.

Evidence that larger suppliers are more likely to specialise in higher fashion products and perform more value-added functions differs from the conclusions drawn in previous research in Lesotho and Kenya (Morris and Staritz 2017; Morris et al. 2016). Moreover, contrary to these studies, we do not find any evidence that larger firms managed by ‘transnational investors’ are less likely to establish up- or downstream linkages with local businesses. In fact, our data show that each supplier interacts on average with one other local firm (either through the outsourcing or sub-contracting of specialised functions), and that this does not vary by investors’ origins.

### Differences in Social Upgrading Before Covid-19

**Fig. 5:**
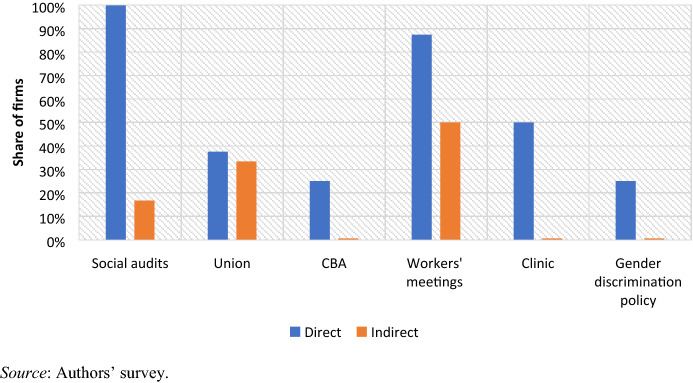
Social upgrading among direct and indirect suppliers (2019)

Direct and indirect suppliers differ significantly on a number of indicators associated with social upgrading (Fig. 5).

First, direct suppliers report undergoing regular social audits—although, as observed elsewhere (Pasquali et al. 2020), these are less rigorous than those previously done by or for US retailers. Fewer than 20% of indirect suppliers reported undergoing audits that included a labour component. As reported by the manager of an indirect supplier operating for a Durban-based design house and producing clothing for a large South African discount retailer:

‘The agent comes fortnightly to inspect quality. They have a QA person based here. They do not inspect anything to do with workers; that is the government’s job.’ (F8, Matsapha, 24 July 2019)

Second, over 60% of firms across both groups have not recognised a trade union;^10^ yet, as reported by a representative of ATUSWA, the number of firms doing so has been slowly increasing over the past few years (F9, Manzini, 15 July 2019). As of 2019, six firms had recognised ATUSWA and two firms (direct suppliers) had signed a collective bargaining agreement (CBA) with the union. All other manufacturers only conformed to the minimum labour conditions as provided by the employment and labour legislation.^11^ The legislation also provides that an employer with more than 25 workers may establish a ‘works council’. All firms reported having either a works council or similar committee through which workers could engage with management (either established by the employer or by the union). However, while all but one direct supplier held regular meetings with the works council, in 50% of indirect suppliers, meetings were less regular and at the management’s discretion.

Third, direct suppliers are more likely to have a clinic in the factory providing workers with free or subsidised healthcare services. As a result of their comparatively small size and capital endowments, indirect suppliers often rely on nearby public hospitals, mobile clinics (co-funded by a number of NGOs active locally), and in-firm first-aid support. Fourth, despite women accounting for over 90% of the workforce, no firm had a gender committee as of 2019, but two direct suppliers (25%) reported having a gender discrimination policy in place.

Importantly, we did not find any significant difference in a number of social upgrading indicators. This includes the share of the workforce that is permanent as opposed to temporary, where the average varies between 78% and 82% across direct and indirect suppliers, respectively.^12^ All suppliers but one were paying strictly at the minimum wage rate and complying with the minimum labour conditions as provided by employment and labour law (at ZAR 418 per week).^13^ Bonuses on productivity targets are paid by most suppliers, while only one firm rewards loyalty through bonuses linked to the length of a worker’s employment at the company. Similarly, all suppliers experience high labour turnover rates of around 30% per year. Moreover, local (Swazi) managers (excluding supervisors) remain a minority in both groups (25% and 24% among direct and indirect suppliers, respectively), while foreign ownership (beyond the SACU region) is also close to 50% in both groups.^14^

Overall, our data show that, pre Covid-19, direct suppliers experienced comparatively higher levels of economic and social upgrading compared to indirect suppliers. Therefore, private governance has played an important role in shaping outcomes for suppliers participating in RVCs. In the section below, we discuss how this difference affect the uneven impact of Covid-19 on Eswatini’s apparel sector.

## Eswatini Apparel Value Chain During Covid-19

The first Covid-19 case was registered in Eswatini on 14 March 2020, with the number of positive patients growing daily over the next two months. Following WHO recommendations, the government enacted a partial lockdown on 26 March, which lasted for six weeks and included the temporary closure of non-essential business and productive activities, the closure of the country’s borders to foreigners, heightened screening measures for freight, the banning of non-essential internal travel, and the suspension of all commercial flights (Gardaworld 2020). The lockdown was partially relaxed on 8 May for productive activities, which enabled most non-essential manufacturing plants to resume activities under stringent health and safety measures and social distancing, including allowing only 50% of the workforce into the factory at any time. Some services (including retail clothing stores) were also allowed to reopen for up to three days a week (Worldaware 2020).

In the apparel sector, the concept of ‘essential production’ extends to cover the manufacture of PPE material and infant clothing, which enabled some manufacturers to produce and export these goods over the period of partial lockdown. After 8 May, however, most firms were open and exporting, though at lower capacity. Critical in this respect has been the relaxation of lockdown measures in South Africa, the main export market for Eswatini’s apparel. South Africa introduced a complete lockdown on 26 March, which included the closure of retail stores and borders, effectively stopping all imports with the exception of essential goods—for apparel this meant only PPE could be imported.^15^ On 1 May, the South African government partially eased the lockdown, moving to Level 4 and enabling the trade and sale of infants’ wear, bedding and winter wear for adults. This move allowed Eswatini’s plants to resume exports. On 1 June, South Africa moved to Level 3, opening up the trade and sale of all clothing categories (Government of South Africa 2020).

As of July 2020, the situation for the country’s apparel export sector was critical, with three plants still closed and a large majority of manufacturers operating at about 50% of their capacity (ETATA, online, 5 June 2020). The situation has been further exacerbated by the heavy toll the lockdown has taken on South African apparel retailers, in particular Edcon, which officially filed for business rescue in April after failing to pay its suppliers for the previous two months (CNBC 2020). Before the Covid-19 outbreak, eight Eswatini apparel exporters were supplying Edcon (at varying volumes), exposing themselves to potentially permanent labour retrenchment and even closure.

### The Impact of Covid-19 on Economic up- and Downgrading

**Fig. 6:**
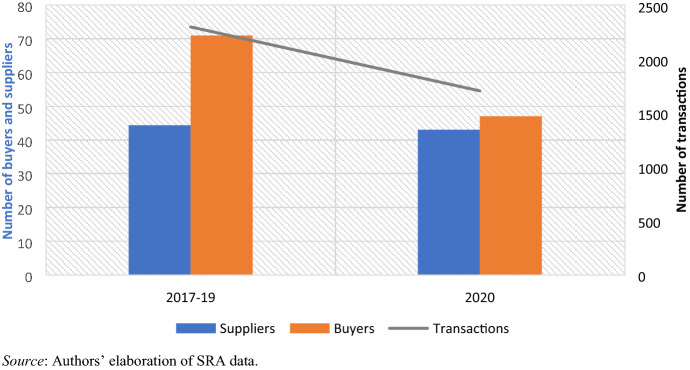
Number of apparel buyers and suppliers and total transactions (March–April)

In order to determine the impact of Covid-19 on economic up- and downgrading we first examine aggregate data for the Eswatini apparel export sector extrapolated from SRA customs data (Fig. 6). The total number of exporters in 2020 does not appear to have changed compared to previous years, but the number of businesses sourcing apparel from Eswatini dropped by about 34%. As a consequence, the data also show a 26% drop in the total number of transactions, i.e., orders. Whereas in the March–April period in 2017, 2018 and 2019 there were on average 2299 export transactions by apparel firms, the Eswatini Revenue Authority recorded only 1705 transactions in the same period of 2020. Regarding the destination of exports, we do not observe any major change, with South Africa remaining the main destination for 95% of the country’s exports. In terms of value, Eswatini exported an average of ZAR 461 million in the March–April period in 2017, 2018 and 2019. In 2020, however, this figure dropped by 46% to ZAR 249 million, reflecting the disruptive impact of Covid-19 on one of the country’s largest export sectors.

From the dataset, we further isolate suppliers that exported before and during the Covid-19 lockdown (i.e. suppliers that exported at least once in the 2017–19 period and once in the 2020 period). To further exclude small and occasional traders, we retain only observations for firms that exported at least ZAR 1 million worth of apparel over the 2017–19 period. This results in a sample of 18 suppliers. We then compute their average annual exported value, average log unit values, and number of exported products for the period before (2017–19 moving average) and during the lockdown (March–April 2020) period. In doing so, we separate the top six firms and the bottom 12, based on their share of exports in 2017–19 (Table 3, Appendix).^16^ As noted previously, the top six firms are all large exporters directly contracted with South African retailers, whereas the remaining exporters operate mostly through design houses.^17^

**Fig. 7:**
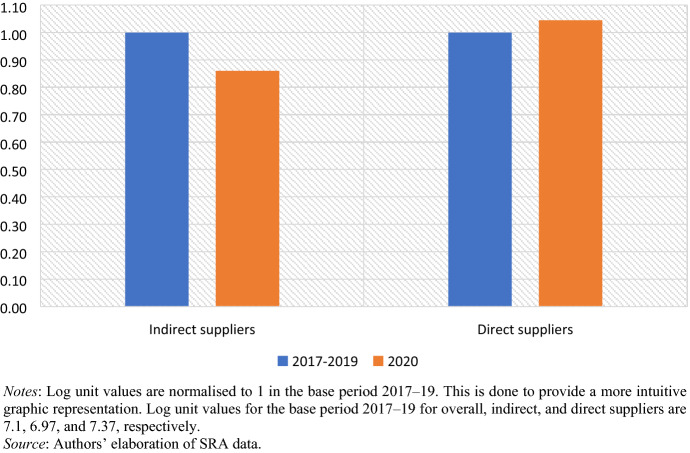
Apparel suppliers’ average unit values—normalised to 1 in 2017–19 (March–April)

In terms of average exported value, both direct and indirect suppliers experienced a significant drop. For the latter, this drop averaged -35% compared to their yearly exports in the March–April period in 2017–19. For the former the drop was even greater, at − 42% compared to their exports in the years predating Covid-19.^18^ When it comes to unit values, direct suppliers reported a marginal increase of 4.5%. Conversely, indirect suppliers suffered an average drop of 14% in March–April 2020 compared to the same period in 2017–19 (Fig. 7). Arguably, this evidence suggests that, while direct suppliers have reduced their total exports without dropping their overall unit costs, smaller CMT plants have engaged in cost-driven competition, dropping prices to retain orders. This conclusion is supported by the comments of the chair of the Eswatini Textile and Apparel Traders Association (ETATA), who is also the owner of a direct supplier: ‘We have been doing business for 15 years with this buyer and we have a good relationship. They work with us, they do not squeeze us. However, I observed that other companies that do business with those on the other side in Durban [design houses], they are being squeezed and buyers try to get them to sell the same products at lower prices. Other firms in ETATA complained about this’ (online, 4 June 2020). Furthermore, while none of the five direct suppliers that responded to our online questionnaire indicated any price reduction by buyers, the opposite is reported by two of three relatively small firms classified as indirect suppliers: ‘The buyer has reduced orders and prices, I am now working at a loss…This cannot continue’ (F1, Manzini, 12 July 2019).^19^

**Fig. 8:**
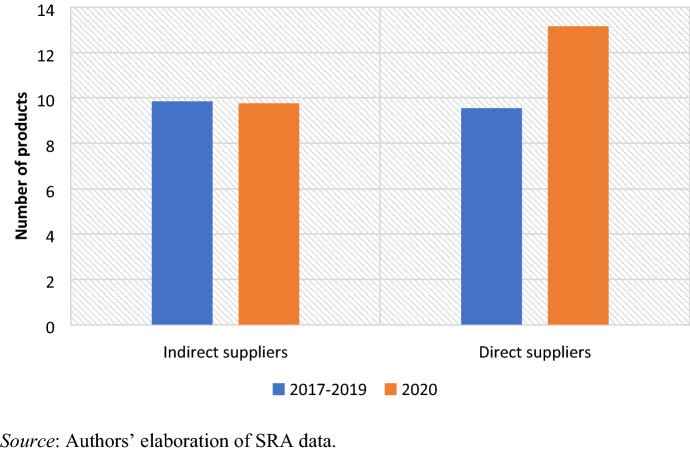
Apparel suppliers’ average number of exported products (March–April)

Finally, the average number of exported products did not change for indirect suppliers. Conversely, direct suppliers considerably broadened their basket of products from an average of 9.5 to 13—an increase of 37% (Fig. 8). This is doubtless the consequence of some firms converting part of their work to the production of personal protective equipment (PPE). Four out of five direct suppliers introduced (or increased) PPE production for the domestic and (in two cases) the South African export market. As the manager of a direct supplier producing PPE reported: ‘We got an opportunity to get into producing PPE and face masks and that allowed us to keep working, first for the local market and recently also for export to South Africa, as our main buyer won a tender with the government there’ (F2, online, 6 June 2020).

Overall, we observe that the Covid-19 pandemic and the initial responses by the governments of Eswatini and South Africa translated into a loss of income for Eswatini’s apparel suppliers in terms of total sales and unit prices. Economic downgrading has therefore occurred. This has, however, been different across direct and indirect suppliers, with the latter experiencing a significantly more severe drop in unit prices. Furthermore, large direct suppliers have also differentiated their product basket significantly more than their counterparts, moving into the production of PPE equipment for the domestic and export markets.

### Impact of Covid-19 on Social Up- and Downgrading, and the Role of Public Governance

Sectoral data on employment during and following the Covid-19 lockdown is not available. Based on information provided by employers during our fieldwork in 2019, we recorded a total of 21,768 workers for the sector, of which 18,000 were in permanent employment. This is very close to the figure of 22,000 directly employed workers provided by ETATA and the Eswatini Investment Promotion Agency (EIPA). Interviews with key stakeholders in 2020, as well as data provided in response to our online questionnaire, suggest that, as of July 2019, the entire workforce remained employed. But the hours worked and income earned had declined significantly.

Three relatively small indirect suppliers (with about 1000 employees in total) had not resumed operations since the beginning of the lockdown in March, and it is possible that these firms will close permanently, with the workers being retrenched. In June–July 2020, most of the other factories appeared to be operating at about 50% capacity, with the labour force split in half and working on rolling shifts (daily, weekly and fortnightly). Workers were paid only for the time they worked, so their income was about half of what they were earning at the start of 2020—i.e. machinists were earning ZAR 900–1000 a month (about US$52–59), significantly below the sector’s minimum wage and the country’s living wage.^20^

As of July 2020, despite a pledge to support retrenched workers with ZAR 25–30 million, the government had not delivered on its promise. While workers in compliant apparel plants in neighbouring South Africa received full pay for the initial six week lockdown period through the Unemployment Insurance Fund (UIF), Eswatini managers and ATUSWA, the major trade union in the sector, complained of a complete lack of support from the Eswatini government (Government of South Africa 2020).^21^ The General Secretary of ATUSWA stated: ‘Since the easing of the lockdown, most factories have re-opened and are working with 50% of the staff at factory and with social distancing measures. However, workers are being paid for the time worked only, effectively earning 50% of their normal wage’ (online, 5 June 2020). The Chair of ETATA, who currently represents the sector in the committee for the Post-Covid-19 Economy Recovery Plan, warned against the potential consequences of government inaction: ‘Look at what the South African government has been doing for the textile industry…We are trying hard to explain to the government how much this industry contributes to the Kingdom. I estimate that this crisis could translate into a permanent loss of 30–35% of the workforce if nothing is done’ (online, 4 June 2020).

Eswatini’s Employment Act provides for wage security. When starting a business, each employer must pay to government the equivalent of one month’s wage for each of its employees, which is held in a trust to pay employees if the firm endures financial hardships. However, payment of this security is at the discretion to the Labour Commissioner, who can exempt an employer from making the payment. According to a key informant, many of the apparel firms were given exemptions, leaving the trust almost empty: ‘We found that in fact very few apparel manufacturers had paid into the fund and to all intents and purposes there is no money available in this fund’.

A second source of funds that the government could tap into is the National Provident Fund (SNPF). Workers and employers must each contribute 5% of the gross weekly wage to the SNPF. Discussions between the government and employers about accessing some of these funds have been ongoing, with some employers having formally applied to lay off workers for the legal maximum of 14 days.^22^ However, the amount being proposed would provide only ZAR 230 per month to each worker, which is well short of the amount needed to sustain a household. Crucially, ATUSWA has been largely ignored in the negotiations and, as reported by their general secretary, only three firms consulted (separately) with the union about lay-offs.

Factory managers are also critical of government over its lack of support for the sector and its general mismanagement of the crisis:

We as management and staff are fully aware of the dire consequences if utmost care is not taken proactively in combatting this dreaded disease… Sadly, our measures are not supported by the government, as during the whole Covid-19 the various ministries charged with this responsibility still remain absolutely silent and shone in their absence. Cases are on the rise and while everything is done by us to combat this disease infections are a real possibility and will have as consequence the shutdown of the factory with more lost production. (F3, online, 12 June 2020)

Employers, however, are not blameless. The Regulation of Wages (Textile and Apparel Industry), issued in terms of the Wages Act of 1964, provides that an employer may lay off workers without pay, for reasons or circumstances beyond his or her control, for a period of up to 14 days. At the end of this period the employer must either re-employ the workers in their original jobs or terminate their employment with notice. We were not told of any firms that had complied with this provision. Instead, workers were left in limbo, neither formally laid off nor retrenched, which meant that they did not receive wages or severance pay, nor could they apply for their provident fund benefits from the SNPF.

With the exception of Edcon, no other retailer has refused to pay for orders placed, in process or shipped. Furthermore, unlike design houses, no retailer has tried to renegotiate prices downwards: ‘They [buyers] have been paying on time, we cannot complain’ (F4, online, June 2020). This statement is echoed by all direct suppliers who responded to our questionnaire. Three direct suppliers further noted that retailers had provided them with some forms of financial assistance: ‘They assisted me with 80% payment for the staff while I was shut down. They asked me for a list of the staff, and they accepted to pay 80% while I was shut. They are also supporting me now by commissioning masks for South Africa’ (F5, online, 4 June 2020). Similarly, the manager of a large FOB direct supplier emphasised: ‘Fabric suppliers are [taking] two weeks longer than before and customers’ selling performance is not stable yet. Our main buyer gave us a loan of ZAR 5 million to buy fabric’ (F6, online, 12 June 2020) Importantly, therefore, we notice that private governance by South African retailers has provided some form of support to direct suppliers.

## Conclusion

**Fig. 9:**
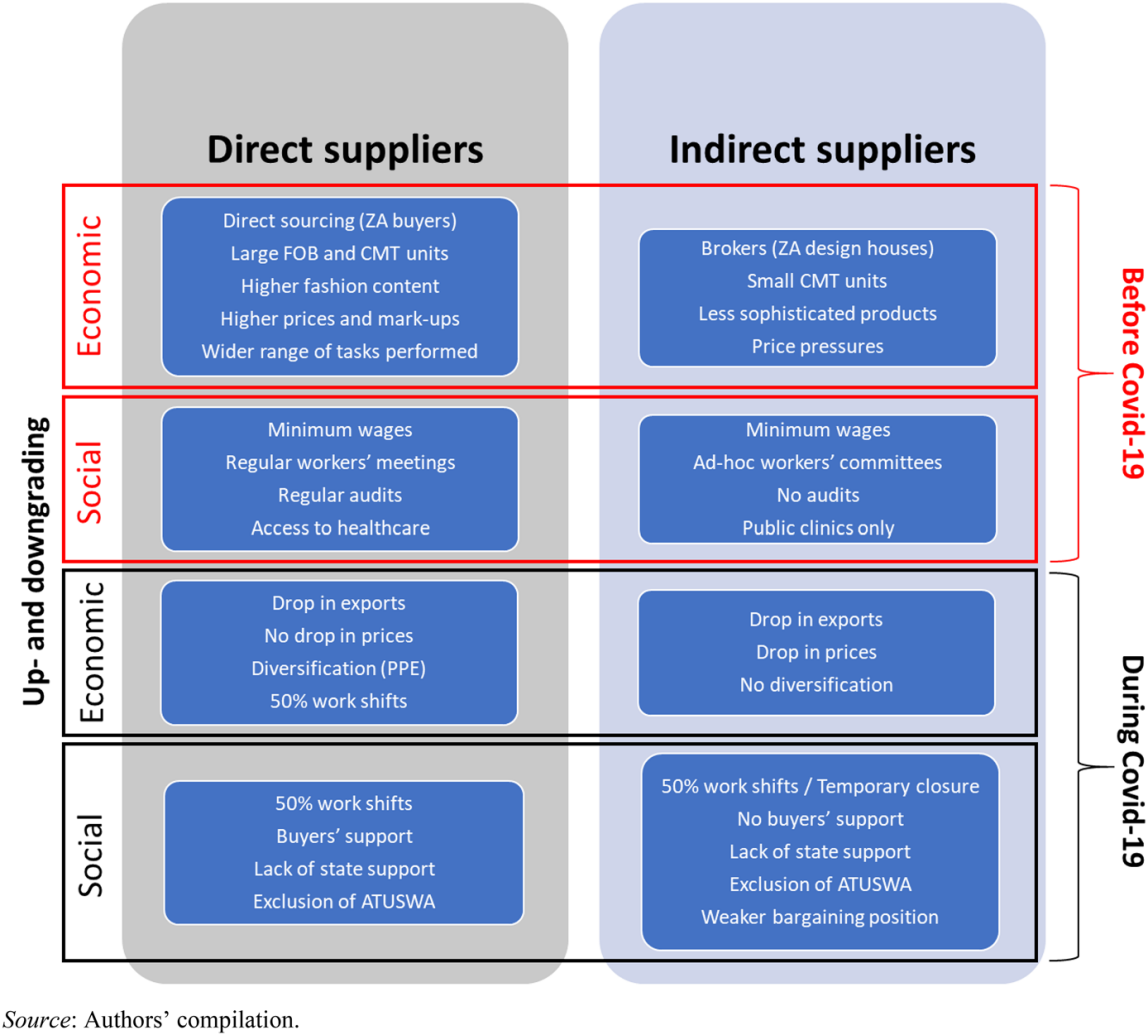
Eswatini apparel suppliers’ economic and social upgrading and downgrading before and during Covid-19

In this article we have explored the governance of Eswatini’s apparel value chain with South Africa and the initial impact that Covid-19 has had on the economic and social up- and downgrading of firms and workers. A summary of the situation is shown in Fig. 9. Interviews conducted in 2019 with managers at all apparel manufacturers in Eswatini revealed that the private governance of apparel RVCs presents two structures: one entailing direct contracting between large suppliers and the South African retailers; the other characterised by indirect supply to the South African market through design houses, which in turn contract with the retailers.

An important finding from our fieldwork in 2019 is that large direct suppliers experienced relatively higher levels of economic and social upgrading. Notably, direct suppliers are comparatively more likely to integrate a larger number of production functions, be regularly audited, produce more complex items with higher mark-ups, and contribute more in terms of taxation. Furthermore, while almost all firms pay at the minimum wage rate, direct suppliers are more likely to undergo social audits, hold regular workers’ meetings, and provide workers with access to basic healthcare facilities. This result underscores the importance of private governance in shaping firms’ differential paths of economic and social upgrading.

A second key finding from primary data gathered in 2020 (including transaction customs data and questionnaire responses), is that private governance has critically determined the uneven impact of Covid-19 on Eswatini’s apparel suppliers and their workforce. While all firms experienced economic and social downgrading, indirect suppliers and their workers have been relatively worse off. The pandemic’s impact was therefore influenced by the private governance of the value chain, with those firms (and their workers) in weaker bargaining positions suffering the most. This finding is unsurprising. The global financial crisis of 2008–10 also led to consolidated relationships between lead firms and first-tier suppliers, often resulting in second- and third-tier suppliers being cut out of the value chain or having their bargaining position significantly undermined (Staritz et al. 2011; Gereffi 2014).

The third finding is that public governance by the Eswatini state has been ineffective in its response to Covid-19, failing to provide any support to the apparel sector and, in particular, to workers. This is in part because developing countries’ governments often have limited resources and restricted fiscal room to manoeuvre (Kjaer et al. 2015; Tyce 2019). It is the case, for example, that earlier decisions by the Eswatini state to favour foreign investments by exempting investors from paying the required wage security are now having serious consequences for workers in the sector, who cannot rely on such funds. The state’s ineffective response, however, has been compounded by its autocratic nature, which has seen it largely ignore statutory provisions regarding lay-offs, to the detriment of workers, and exclude ATUSWA from the deliberations about relief measures. These findings underscore previous studies suggesting that, in the absence of strong public policy, private governance is not up to the task of dealing with the impact of a shock of the scale of Covid-19 (Anner 2020b).

Compared to the situation for global value chains, however, Eswatini has come off relatively lightly. In Bangladesh, for instance, European and US lead retailers cancelled over 70% of their orders and refused to honour their contractual obligations for orders already in transit, leading to the shutdown of factories within weeks of the beginning of the crisis (Anner 2020a). By contrast, with only one exception, all South African buyers have honoured their contractual agreements with Eswatini suppliers and, in a few cases, have provided them with direct support. This suggests some support for the argument made in recent research that ‘the Covid-19 pandemic has highlighted the falsity of any assumption that the global North has all the solutions to tackle global challenges’ (Oldekop et al. 2020, forthcoming).

The finding that private governance structures have an important influence on upgrading trajectories is an important contribution to the theorisation of value chain governance. However, it was not unexpected that Covid-19 would have a differential impact along this same fault line, nor that the pandemic would lead to economic and social downgrading for all suppliers and their workers. Similarly, the weak response of the poorly resourced Eswatini government is not surprising. What is exceptional and needs further explanation is the position taken by the major South African retailers, which sets them sharply apart from their US and EU counterparts in Bangladesh (Anner 2020a, b). This is especially since almost all South African retailers have been shown to maintain little or no interest in corporate social responsibility (including private codes of conduct and social auditing of suppliers) (Pasquali et al. 2020).

We did not explore the nature of this relationship in detail so we can draw only tentative conclusions. One explanation is that direct contracting between retailers and suppliers has established a qualitatively different relationship to the one-step-removed ‘relationship’ that exists where there is in intermediary. The distinction in these types of relationship would depend less on the sort of technical criteria that Gereffi et al. (2005) used to identify modular, relational and captive forms of governance and more, we would argue, on territorial proximity and embeddedness (Hess 2004; Morris et al. 2016). Another (related) explanation is that being in the regional neighbourhood elicits a greater sense of shared economic and social destiny in the context of major shocks, which for retailers opposes decisions motivated by short-term profitability. Ultimately, South African retailers need markets in the region to remain viable, which their actions vis-à-vis suppliers in less developed neighbouring countries can contribute to or undermine. Both of these explanations are rooted in a paradigm of regional proximity that is not applicable to North-South apparel GVCs. Clearly, however, more research on this issue is required.

## Appendix: Online questionnaire (July 2020)

1. What are the main challenges your business is facing with the present Covid-19 crisis?
2. Please select the right answer:
  - Orders have continued as normal.
  - Buyers have stopped new orders but paid for orders already in production.
  - Buyers have stopped new orders and cancelled orders in production (without paying).
  - Buyers have sent back orders in transit (without paying).
  - Buyers have reduced prices.
  - Other.
3. Have any of the buyers provided you with a support scheme to get through the lockdown?
  - If yes, what type of support did you receive?
4. Have buyers taken advantage of the crisis to reduce prices?
  - If yes, by how much (%)?
5. Are you now producing PPE equipment?
  - If yes, for whom?
  - If not, why not?
6. Has the government provided any support to your firm or workers?
7. How many workers have you retrenched since March 2020?
  - Did you pay these workers severance pay?
  - If yes, how much of their monthly salary?
8. How many workers have been put on part-time/reduced shifts and for what period?
9. Do you think your factory will still be open in one year from now?
10. Is there anything else you would like to say about how your firms is dealing with the crisis?

See Tables 1, 2 and 3.

**Table 1:** Intra-Africa apparel exports by main exporting countries: 2010–18 (US$000)

	2010	2011	2012	2013	2014	2015	2016	2017	2018	2019
Eswatini	62,631	94,874	118,872	128,805	153,938	154,868	168,448	206,531	216,513	218,268
Lesotho	47,929	61,972	64,261	75,149	96,421	104,103	116,593	137,306	164,555	169,476
Mauritius	77,859	111,627	152,243	151,566	136,464	133,566	113,928	122,512	144,386	143,075
Madagascar	20,395	41,906	65,006	88,620	92,203	102,762	98,556	91,777	102,336	97,425
Kenya	33,482	33,684	46,615	36,720	45,659	37,587	34,589	27,710	25,204	32,710

*Source* UN-COMTRADE

**Table 2:** Eswatini’s apparel export destinations by value (US$000)

	2010	2011	2012	2013	2014	2015	2016	2017	2018	2019
South Africa	62,423	94,389	118,164	128,118	153,214	153,339	167,631	205,259	214,350	216,696
USA	97,841	80,200	62,398	51,870	57,379	2683	1011	410	1059	6
Others	1131	2274	3524	1760	1579	2594	1827	3422	4818	3999

*Source* UN-COMTRADE

**Table 3:** Suppliers’ export share in the period 2017–19

	Supplier	Export share (2017–19) (%)
1	SRA 22852	28.00
2	SRA 47817	16.02
3	SRA 35805	13.76
4	SRA 13940	9.87
5	SRA 34718	9.55
6	SRA 44270	8.33
7	SRA 17680	2.69
8	SRA 33645	2.06
9	SRA 50004	1.62
10	SRA 26529	1.32
11	SRA 21061	1.22
12	SRA 50151	1.17
13	SRA 42395	1.00
14	SRA 30864	0.95
15	SRA 26967	0.71
16	SRA 97003	0.68
17	SRA 21604	0.46
18	SRA 50262	0.38

*Source* Authors’ elaboration based on SRA data

The table excludes firms that do not appear as registered exporters in 2020

## Footnotes

1 The authors further refer to a category of ‘indigenous investors’, yet this category is limited to Mauritius and Madagascar, while ‘Swaziland [Eswatini] and Lesotho have no significant indigenous owned apparel exporters’ (Morris et al. 2016, p. 1251).

2 Eswatini was previously referred to as Swaziland until 2018. The union ATUSWA has retained the old acronym.

3 Lubatkin et al. (1993) found a strong correspondence between product-count measures and Rumelt’s (1974) categorical measures of diversification, which supports the validity of product-count measures.

4 A major limitation of this paper is that we did not interview workers in 2020. Our information on labour (post-Covid-19) is based solely on firms’ online responses and on interviews with the General Secretary of ATUSWA and the Chair of the Eswatini Textile and Apparel Traders Association (ETATA) in June 2020.

5 Percentage based on UN-COMTRADE dataset (https://comtrade.un.org). The data excludes South Africa’s exports, since over 95% of these are re-exports (Barnes and Hartogh 2019).

6 Together, these six retailers account for over 90% of the South African internal market (Barnes and Hartogh 2019) and have recently begun expanding across Africa and globally (Pasquali et al. 2020).

7 Two firms operate via both direct contracts and indirectly through design houses.

8 One firm operates exclusively for the domestic market under its brand; it is therefore excluded from the analysis.

9 As of 2019, Pepkor is the only retailer sourcing in Eswatini almost exclusively through design houses. Mr Price and Edcon maintain a mix of direct and mediated interactions.

10 Recognition occurs when the employer formally acknowledges a trade union as the representative of the workforce within the factory. In Eswatini recognition is mandatory when 50% or more of the workforce are paid-up members of the trade union.

11 One firm recognises a different and smaller union, Swaziland Industrial Workers Union (SIWU).

12 All firms follow the national labour legislation, which allows an employer to employ a worker for a probation period of up to three months before taking them on permanently.

13 Eswatini’s minimum wages in the apparel sector are among the lowest in the region, about a third of those in South Africa and below Lesotho’s minimum wage (Pasquali et al. 2020).

14 Foreign (non-SAUC) owners are mainly Taiwanese investors as well as one Filipino and one from Hong Kong. There are also 3 Swazi entrepreneurs, while the rest is South African.

15 See South African Government Gazette, 25 March 2020, for a list of exemptions during the lockdown.

16 The top six suppliers have a significantly larger market share across the 2017–19 period. Table 1 in the appendix reports the market share of the top 18 exporting firms, with the largest gap between supplier number six and seven. Nevertheless, extending the upper quartile to include the top seventh and eighth exporters holds consistent results. These numbers are consistent with the structure of the Eswatini apparel industry, where the top six firms account for about 80% of the market share and employ over 1000 employees each (data matched with authors’ 2019 survey).

17 To the extent that data are anonymised, and we cannot directly match firms to data, we base this statement on the evidence derived from Figure 3 that larger firms are also direct suppliers.

18 The firm’s yearly average exported value for the base period 2017–19 for indirect and direct suppliers are ZAR 23.5 million and ZAR 58.7 million, respectively. In 2020, the same values drop to ZAR 13.8 and 33.6 million, respectively.

19 While direct suppliers did not report issues linked to the importation of fabrics, smaller indirect CMT suppliers do not control this stage of the value chain, which is performed by *design houses*. As we lack data from design houses, we cannot identify the exact cause of lowering unit values and whether this is linked to difficulties experienced in the procurement stage.

20 Legal Notice No. 51 of 2018, under the Wages Act of 1964 (Act No. 16) establishes the minimum wages for the textile and apparel sectors (under section 11). Permanent machinists’ minimum wage is set at ZAR 418.18 per week, while the wage for casual labourers is ZAR 283.72. These wages are significantly below the monthly living wage for Eswatini of ZAR 3,076 per month (SEPARC 2016).

21 Some reservations have been expressed about whether or how quickly UIF benefits have reached apparel workers in South Africa (Business & Human Rights Resource Centre 2020). Furthermore, it should be noted that UIF benefits only went to firms that are registered and compliant with the National Bargaining Council for the Clothing Manufacturing Industry (NBCCI). Previous research has shown the existence of high levels of non-compliance in South Africa’s apparel industry (Godfrey 2013).

22 We do not have data on the number and type of firms that applied for lay-offs.
