# Correction to: Governance of Eswatini Apparel Regional Value Chains and the Implications of Covid-19

**DOI:** 10.1057/s41287-021-00392-2

**Published:** 2021-04-15

**Authors:** Giovanni Pasquali, Shane Godfrey

**Affiliations:** 1 The Global Development Institute, University of Manchester, Arthur Lewis Building, Room 2.037, Oxford Road, Manchester, M13 9PL UK; 2 Institute of Development and Labour Law, University of Cape Town, Cape Town, South Africa

## Correction to: The European Journal of Development Research 10.1057/s41287-021-00383-3

### Acknowledgements

We would like to thank the ESRC-GCRF for the ES/S000453/1 grant to which this paper is linked as part of the project ‘Shifting South: decent work in regional value chains and South-South trade’. We are also extremely grateful to the Eswatini Revenue Authority for the collaboration, and the four anonymous referees and the editors for the insightful comments which have significantly improved the paper. Finally, the completion of this work has benefitted from the useful comments of Prof Stephanie Barrientos and our colleagues of the GPN Research Group at the University of Manchester.

